# Rates of Litter Decomposition and Soil Respiration in Relation to Soil Temperature and Water in Different-Aged *Pinus massoniana* Forests in the Three Gorges Reservoir Area, China

**DOI:** 10.1371/journal.pone.0101890

**Published:** 2014-07-08

**Authors:** Wenfa Xiao, Xiaogai Ge, Lixiong Zeng, Zhilin Huang, Jingpin Lei, Benzhi Zhou, Maihe Li

**Affiliations:** 1 State Forestry Administration Key Laboratory of Forest Ecology and Environment, Research Institute of Forest Ecology, Environment and Protection, Chinese Academy of Forestry, Beijing, PR China; 2 Research Institute of Subtropical Forestry, Chinese Academy of Forestry, Fuyang, Zhejiang, PR China; 3 State Forestry Administration Key Laboratory of Forest Silviculture, Research Institute of Forestry, Chinese Academy of Forestry, Beijing, PR China; 4 Swiss Federal Research Institute WSL, Birmensdorf, Switzerland; 5 State Key Laboratory of Forest and Soil Ecology, Institute of Applied Ecology, Chinese Academy of Sciences, Shenyang, PR China; Tennessee State University, United States of America

## Abstract

To better understand the soil carbon dynamics and cycling in terrestrial ecosystems in response to environmental changes, we studied soil respiration, litter decomposition, and their relations to soil temperature and soil water content for 18-months (Aug. 2010–Jan. 2012) in three different-aged *Pinus massoniana* forests in the Three Gorges Reservoir Area, China. Across the experimental period, the mean total soil respiration and litter respiration were 1.94 and 0.81, 2.00 and 0.60, 2.19 and 0.71 µmol CO_2_ m^−2^ s^−1^, and the litter dry mass remaining was 57.6%, 56.2% and 61.3% in the 20-, 30-, and 46-year-old forests, respectively. We found that the temporal variations of soil respiration and litter decomposition rates can be well explained by soil temperature at 5 cm depth. Both the total soil respiration and litter respiration were significantly positively correlated with the litter decomposition rates. The mean contribution of the litter respiration to the total soil respiration was 31.0%–45.9% for the three different-aged forests. The present study found that the total soil respiration was not significantly affected by forest age when *P. masonniana* stands exceed a certain age (e.g. >20 years old), but it increased significantly with increased soil temperature. Hence, forest management strategies need to protect the understory vegetation to limit soil warming, in order to reduce the CO_2_ emission under the currently rapid global warming. The contribution of litter decomposition to the total soil respiration varies across spatial and temporal scales. This indicates the need for separate consideration of soil and litter respiration when assessing the climate impacts on forest carbon cycling.

## Introduction

Soil respiration is a major process controlling carbon (C) loss from terrestrial ecosystems [1]. Globally, soil respiration releases approximately 80 Pg C into the atmosphere per year [2], which is estimated to account for 20–38% of the total annual biogenic CO_2_ emissions to the atmosphere [3]. Given the predicted increase in atmospheric CO_2_ concentrations, litter as a main C source for the total soil CO_2_ efflux will increase [4]. Litterfall represents a major flux of the vegetative C to soil, and hence, changes in litter inputs are likely to have wide-reaching consequences for soil C dynamics [5]. Decomposition plays an integral role in determining soil C entering atmosphere [6], [7], and the fate of C contained in litter, therefore, plays an important role in the long-term C sequestration in forest soils [8], [9]. The total respiration together with litter inputs data could be used to evaluate C dynamics in soils [10], [11]. To better understand and predict the soil C dynamics and cycling in terrestrial ecosystems in a changing world, studies of the relationships between soil respiration and litter decomposition in response to environmental changes are still required.

The aboveground plant litter fluxes have been found to be strongly correlated with soil respiration, and litterfall manipulations have strong effects on soil CO_2_ efflux [12]. Several studies have investigated the contribution of the leaf litter layer to soil respiration [4], [13], [14], [15]. Raich [2] showed that soil C flux from respiration was 2.8–3.0 times higher than the C flux from the aboveground litter production. Buchmann [16] found that the annual C loss through soil respiration (710 g C m^−2^ yr^−1^) was higher than the C input of the annual aboveground litterfall (240 g C m^−2^ yr^−1^). Prévost-Bouré et al. [4] indicated that addition of fresh litter significantly increased the total soil CO_2_ efflux. Sayer et al. [5] indicated that soil respiration was on average 20% lower in the litter removal and 43% higher in the litter addition treatment compared to the controls.

On a global scale, soil respiration is mainly controlled by temperature and precipitation [17]. Many studies have shown that soil temperature is the primary factor determining the rates of soil respiration [18] and litter decomposition, and thus, soil warming increased the decomposition rate of fine woody debris in temperate forests [19]. However, Yuste et al.[17] found that soil respiration was insensitive to temperature in temperate maritime forests during late spring and summer when soil water content was limited. Fierer et al. [20] showed that the temperature sensitivity of litter decomposition varied with litter type and the extent of decomposition. These results indicate that soil respiration and litter decomposition depend on climatic variables such as soil temperature and precipitation [21]. It is thus necessary to see whether the temperature sensitivity of soil respiration and litter decomposition keeps consistent in a changing world.

*Pinus massoniana* Lamb. is a native species with high adaptability and tolerance to drought and low fertility soil but with low productivity in the Chinese subtropical regions. *P. massoniana* forests cover an area of ∼2 million square kilometers in China, and are one of the most important vegetation types with key ecological importance in the Three Gorges Reservoir Area [22]. However, the relationships between soil respiration and litter decomposition in *P. massoniana* forests have not yet been studied. Based on long-term litter decomposition studies, Aerts and de Caluwe [23] concluded that the initial litter respiration rates are reliable indicators for long-term litter decomposability. The present study aimed to investigate the linkage of soil respiration and litter decomposition in different-aged *P. massoniana* forests, to provide basic knowledge for assessing the forest carbon budget and dynamics in those forests. We hypothesized that 1) the dynamics of soil respiration are reliable indicators for litter decomposition rate, 2) the soil respiration and litter decomposition have similar sensitivity to temperature, and 3) the co-variant effects of temperature and soil water contents on both litter decomposition and soil respiration are significant in different-aged *P. massoniana* forests.

## Materials and Methods

### Ethics Statement

This work was approved by Zigui National Forest Ecological Research Station. Our study related to litter decomposition and soil respiration did not involve endangered or protected species, and did not damage or destroy the vegetation and animals, although the study sites were located within a protected area for wildlife in the Three Gorges Reservoir Area, China.

### Study site and forests

The study sites were located in Zigui county (110°00′14″–111°18′41″E, 30°38′14″–31°11′31″N), Hubei province, China. The region has a subtropical monsoon climate with mean annual temperature of 17–19°C and mean annual precipitation of 1000–1250 mm mainly occurring between April and September. The forests studied are *P. massoniana* pure stands on Haplic Luvisol soil [24], with *Camellia oleifera*, *Loropetalum chinensis*, *Cotinus coggygria* and *Echinochloa crusgalli*, *Veronicastrum villosulum* in the understorey. The characteristics of the forests/sites were summarized in Table 1. Table 1 showed that the 30-year-old forests had lower mean values of soil organic matter and total nitrogen contents compared to the other two forests.

**Table 1:** Summary of stand and site characteristics measured in summer 2010 (mean ±1 SD; *n* = 3).

	Forests		
	20-year-old	30-year-old	46-year-old
Stand characteristics			
Elevation (m)	950	350	990
DBH (cm)	16.1±1.15	25.3±0.93	33.1±1.09
Height (m)	15.6–19.0	17.5–21.6	18.4–24.8
Stand density (ha^−1^)	800	710	575
Litter layer depth (cm)	4.03±1.59	5.77±0.85	6.34±1.99
Litter litterfall (t·ha^−1^·a^−1^)	3.38±0.07	4.69±0.20	5.60±0.23
Litter standing crop (t·ha^−1^)	9.35±5.14	9.26±2.97	14.05±6.40
Soil characteristics			
Soil depth (cm)	70-100	60-80	70-100
Soil organic matter (g·kg^−1^)	51.90±8.38	19.87±6.78	69.62±11.57
Total nitrogen (g·kg^−1^)	2.36±0.58	1.24±0.18	3.33±0.70
Soil bulk density (g·cm^−3^)	1.13±0.14	1.53±0.08	1.08±0.14
pH value	5.12±0.33	5.43±0.09	4.50±0.15

Note DBH = mean diameter at breast height.

The experiment included 20-, 30-, and 46-year-old *P*. *massoniana* forests each with three spatially separated stands (*n* = 3) at similar elevations (±50 m). Each stand has an area of more than 1 ha. In the center of each stand, a 20 m×30 m plot was established in June 2010. The rates of litter decomposition and soil respiration were determined in each plot.

### Soil respiration, soil temperature and soil water content

Six polyvinyl chloride (PVC) collars (19.6 cm in inner diameter and 10 cm in height) were randomly installed to a soil depth of 5 cm for soil respiration sampling within each plot in July 2010. The litters were kept intact in three of these six collars, and were removed in the other three randomly selected collars (*n* = 3). The first measurement was conducted in August 2010, i.e. one month after the collar establishment. Soil respiration was measured using Li-8100 portable soil CO_2_ flux system (LI-COR Inc., Lincoln, NE, USA) [25]. All soil respiration measurements were made between 08:00 hours and 11:00 hours (local time), to avoid bias of diurnal changes, according to our pilot tests (data not shown). Each measurement usually takes about 12 min. All the PVC collars were installed permanently throughout the experimental period. The soil respiration was measured monthly from August 2010 to January 2012 (i.e. a total of 18 times of repeated measures).

Soil temperature (T_5_) and soil moisture at 5 cm soil depth were recorded adjacent to each soil respiration collar. Soil temperature was measured with a soil temperature probe. Volumetric soil water content (SWC) was measured using a theta probe (EM50, Decagon, USA), which was calibrated to the soil type in the plots following the procedure described by Delta-T (Luan et al. 2011).

### Litter decomposition experiment

Leaf litter decomposition was studied using the standard litterbag technique [26]. In each stand, freshly fallen leaf litters were collected by hand and air-dried in May 2010, and then well-mixed for the decomposition experiment. Sub-samples of air-dried leaf litters were oven-dried at 70°C to constant weight for calculating the conversion coefficient between air-dried and oven-dried leaf. The initial litter quality was analyzed and summarized in Table 2. Table 2 showed that the leaf litter of the 30-year-old stands had higher C/N and lignin/N ratios than the leaf litters of the other two stands.

**Table 2:** Initial substrate quality (mean ±1SD, *n* = 3) of leaf litter in *Pinus massoniana* forests studied.

Initial litter quality	20-yr-old forests	30-yr-old forests	46-yr-old forests
C %	56.25±0.10a	56.54±0.20a	57.21±2.45a
N %	0.98±0.40a	0.74±0.09a	0.85±0.15a
C/N ratio	62.81±20.56b	76.94±9.56a	68.17±14.45ab
Lignin %	34.27±1.31a	34.31±0.42a	34.24±0.16a
Lignin/N ratio	34.89±3.19b	46.63±5.23a	33.49±9.96b

Note: different letters within a row indicate significant difference (*P*<0.05) among the stands.

Twenty grams of air-dried leaf litters were placed in a nylon-mesh bag with 20 cm×20 cm in size and 1-mm mesh. We placed 36 litter-filled bags in each plot in June 2010, and a total of 324 decomposition bags (3 forests ×3 stands ×36 bags/stand) were placed. Litterbags lying flat on the litter layer surface were fastened using steel screens. Freshly fallen litters above the litterbags were removed monthly. Six bags in each stand were retrieved at 90 d (2nd November, 2010), 180 d (2nd February, 2011), 270 d (2nd May, 2011), 360 d (2nd August, 2011), 450 d (2nd November, 2011) and 540 d (31st January, 2012) of field decomposition, respectively. Each litterbag was brushed to remove the external soils and litters, and then the remaining litter was placed in a paper bag and oven-dried at 70°C to constant weight. Litter water content (LWC) was also calculated for each sample.

### Data analysis

The total soil respiration rate was defined as the sum of the litter respiration plus the litter-free soil respiration. According to Sayer et al. [5], soil and litter respiration was calculated using the equation (1)
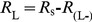
(1)where *R_L_* is the litter respiration, *R_(L-)_* is the litter-free soil respiration (i.e. litter in collar removed), and *Rs* is the total soil respiration. Each *R* was calculated using the equation (2) [3]:
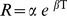
(2)where *R* is the soil or litter respiration, *T* is the temperature at 5 cm soil depth (*T*_5_), *α* is the respiration rate at *T* = 0°C, and *β* is a fitted temperature-response coefficient, according to Rey [27].

The temperature sensitivity (*Q*_10_) of soil respiration (equation 3) [28] and litter mass loss (equation 4) [29] was calculated using the following equations:
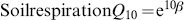
(3)




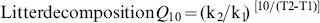
(4)where *β* is a fitted parameter, *k*_1_ and *k*_2_ are the rate constants for a process of interest at two observed temperatures *T*_1_ and *T*_2_.

The co-variant effects of soil temperature and soil moisture on soil respiration were fitted using equation (5), according to Saiz et al. [5].
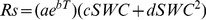
(5)where *R*s is the total soil respiration, *T* is the soil temperature at 5 cm depth, *SWC* is the soil water content at 5 cm soil depth, *a*, *b*, *c*, *d* are fitted parameters.

The decomposition rates were estimated using a single exponential decay model (6) [30]:
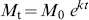
(6)where *M*_t_ is the litter dry mass at time *t*, *M*_0_ is the initial litter mass, *t* is the sampling time interval, and *k* is the annual decay constant.

Repeated measures ANOVAs were used to test the effects of forest age (between subject), time (within subject), and their interaction on soil respiration, litter respiration, litter-free soil respiration, and litter decomposition rate. One-way and Univariate ANOVAs were applied to compare the litter leaf loss rate followed by Tukey's test. Exponential regression analyses were used to examine the relationships between environmental factors (soil temperature and soil water content) and respirations or litter decomposition rates, as well as the relationships between soil respirations and litter decomposition rates. A log-transformed model was applied to calculate the annual decay rate constants (*k*) from litter dry mass remaining. All statistical analyses were performed using SPSS 16.0 software package for windows (SPSS Inc., Chicago, USA).

## Results

### Dynamics of soil and litter respiration

**Figure 1:**
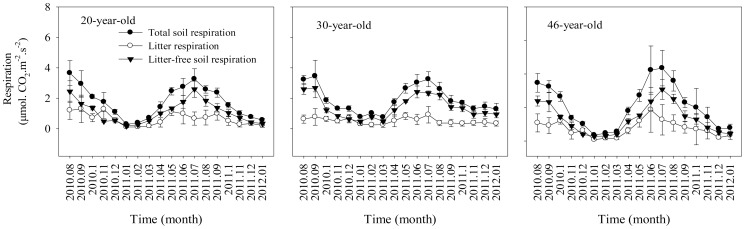
Seasonal patterns and monthly dynamics of the total soil respiration (•), litter respiration (○), and the litter-free soil respiration (▾) in different-aged *Pinus massoniana* forests (mean ±1SD, *n* = 9).

The litter respiration, litter-free soil respiration, and the total soil respiration rates did not vary with forest age across the experimental period (Table 3). Values of all those parameters changed significantly with time, showing obvious seasonality. The forest age effects on litter and soil respiration changed with time, showing significant stand × time interactions (Table 3). The litter respiration had the lowest value in January, and peaked in May/June, whereas the total soil respiration and the litter-free soil respiration had lower rates in winter and higher rates in summer, with the highest rates in August and lowest values in January (Fig. 1). Thus, the highest value of litter respiration occurred nearly one month earlier than the peak values of the total soil respiration and the litter-free soil respiration (Fig. 1).

**Table 3:** Effects of forest age and sampling time on the total soil respiration, litter-free soil respiration, and litter respiration in *Pinus massoniana* forests tested using repeated measures ANOVAs.

	Between subject			Within subject							
	Forests			Time				Forest × Time			
	df	F	*P*	df	F		*P*	df	F		*P*
Across the experimental period											
Total soil respiration	2	0.323	0.725	17	88.023	0.00		34	4.2	0.00	
Litter-free soil respiration	2	0.782	0.463	17	10.401	0.00		34	2.985	0.00	
Litter respiration	2	1.114	0.336	17	120.884	0.00		34	3.873	0.00	
April - October											
Total soil respiration	2	1.215	0.312	9	28.301	0.00		18	2.563	0.001	
Litter-free soil respiration	2	0.669	0.521	9	42.848	0.00		18	2.719	0.000	
Litter respiration	2	5.986	0.007	9	3.826	0.00		18	1.842	0.022	
November - March											
Total soil respiration	2	3.355	0.050	7	25.834	0.00		14	6.190	0.00	
Litter-free soil respiration	2	7.855	0.003	7	12.238	0.00		14	6.043	0.00	
Litter respiration	2	0.541	0.59	7	14.635	0.00		14	7.196	0.00	

The mean annual litter-free soil respiration was 1.47±0.92 (mean±1SD), 1.58±0.83, and 1.68±0.99 µmol CO_2_ m^−2^ s^−1^ in the 20-, 30 and 46-year-old stands, respectively (Fig. 1). The mean litter respiration was 0.81±0.66, 0.60±0.66, and 0.71±0.77 µmol CO_2_ m^−2^ s^−1^ in the 20-, 30-, and 46-year-old stands, respectively (Fig. 1). The litter respiration rates were always lower than the litter-free soil respiration rates, except for some cases in November and December (Fig. 1).

The total soil respiration tended to be somewhat higher (*P*>0.05) in the 46-year-old stands than in the 20- and 30-year-old stands in summer, but it was significantly higher (*P* = 0.05) in the 30-year-old stands than in the other two stands in winter (Table 3, Fig. 1). The total soil respiration rates increased from spring to summer and reached the maximum values of 3–4 µmol CO_2_ m^−2^ s^−1^ in July/August (Fig. 1). The mean total soil respiration across the experimental period was 1.94±1.28, 2.00±1.0, and 2.19±1.51 µmol CO_2_ m^−2^ s^−1^ in the 20-, 30-, and 46-year-old stands, respectively (Fig. 1).

### Litter decomposition and its relationship to soil respiration

**Figure 2:**
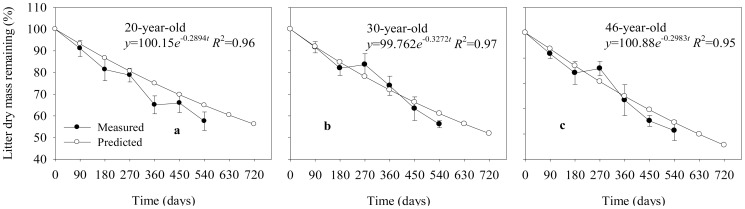
Observed (•) and predicted (○) values of litter dry mass remaining across the experimental period in different-aged *Pinus massoniana* forests (mean ±1SD, *n* = 9).

The leaf litter decomposition rates did not statistically differ among the different-aged stands (data not shown) and followed a similar pattern through the whole decomposition time (Fig. 2). After 540 days' decomposition in the field, the remaining litter ranged from 57.6% in the 20-year-old stands, to 56.1% in the 30-year-old stands, and 61.3% in the 46-year-old stands compared to their initial mass (Fig. 2). The litter decomposition rates were relatively slow and constant during the first six month, and then increased with the onset of the rainy season. The decomposition rates were lower during the dry period from March to June (180–270 d) than those during the hot season in summer.

An exponential model of decomposition was fitted to the experimental data (Fig. 2). The annual decomposition rate coefficients (*k*) were 0.29 (20-year-old stands), 0.33 (30-year-old stands), and 0.30 (46-year-old stands). The predicated remaining litter was 64.9%, 61.1%, and 64.5% in the 20-, 30-, and 46-year-old stands after 540 days of field decomposition, respectively. The predicted data of litter mass remaining slightly overestimated the measured data (Fig. 2). The unexpected higher values of litter mass remaining measured at 270 d in the 30- and 46-year-old stands may be caused by bias with respect to uncontrolled field experimental conditions and to lower decomposition rate during the dry period from March to May (e.g. 180–270 days) (Fig. 2).

**Figure 3:**
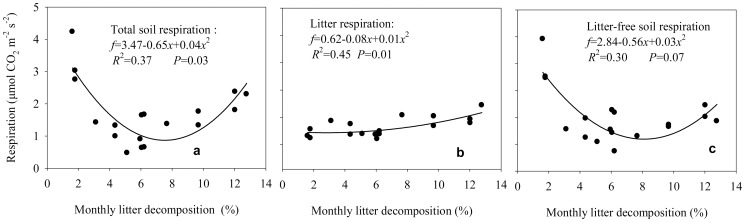
Relationships between mean monthly litter decomposition and the total soil respiration (a), litter respiration (b), and litter-free soil respiration rate (c) for different-aged *Pinus massoniana* forests. Note: respiration rate is the mean value across the litter decomposition period.

The total soil respiration, litter respiration, and litter-free soil respiration in relation to litter decomposition had similar patterns for the three stands, showing a quadratic function (*R* ^2^ = 0.37, 0.45, 0.30, and *P* = 0.03, 0.01, 0.07, respectively; see Fig. 3). The mean percentage contribution of litter respiration to the total soil respiration was 45.9% (ranging from 19.1 to 72.6% across the experimental period) in the 20-year-old stands, 31.0% (14.3–47.6%) in the 30-year-old stands, and 38.3% (15.6–61.0%) in the 46-year-old stands. The contribution of litter respiration to the total soil respiration was larger in winter than in summer.

### Soil respiration and litter decomposition rate in relation to soil temperature and water content

**Figure 4:**
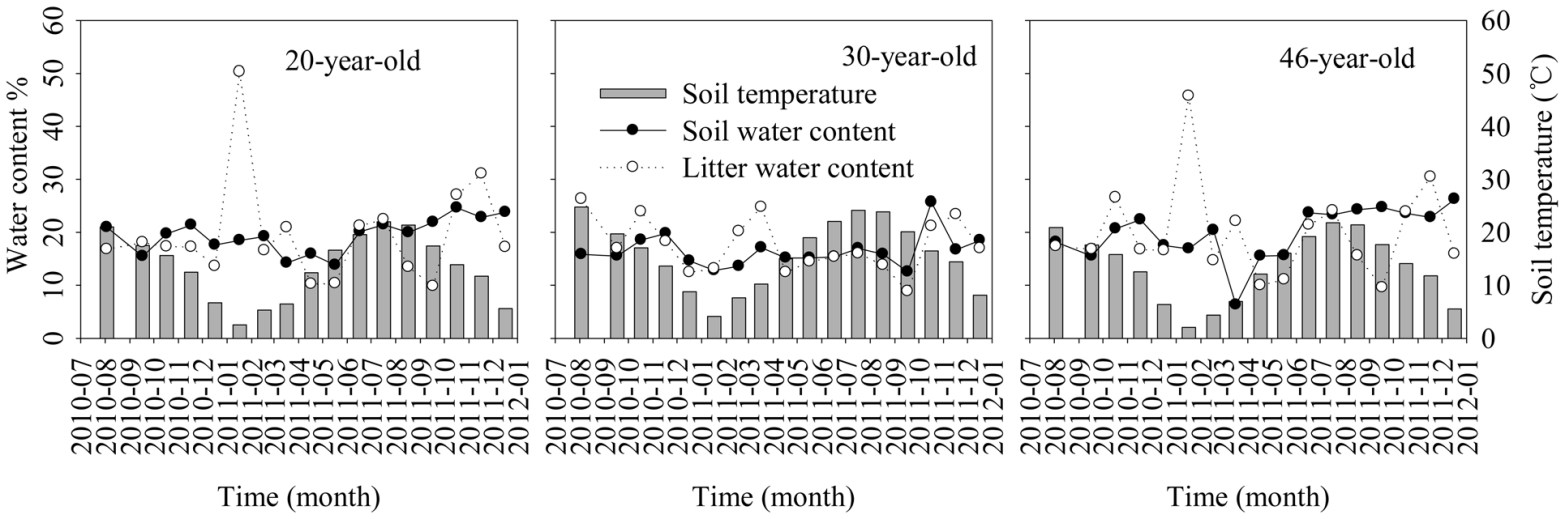
Soil temperature (5 cm soil depth), soil water content (5 cm soil depth) and litter water content in different-aged *Pinus massoniana* forests studied across the experiment period.

The annual mean soil temperature (*T*_5_) was 12.64°C (ranging from 2.40°C in January to 23.60°C in July) in the 20-year-old stands, 15.08°C (2.80 to 25.80°C) in the 30-year-old stands, and 12.83°C (2.70 to 25.53°C) in the 46-year-old stands (Fig. 4). The annual mean soil water content and litter water content (LWC) were 19.70% and 20.86%, 16.28% and 16.70%, 20.29% and 20.36% in the 20-, 30-, and 46-year-old stands, respectively (Fig. 4).

**Figure 5:**
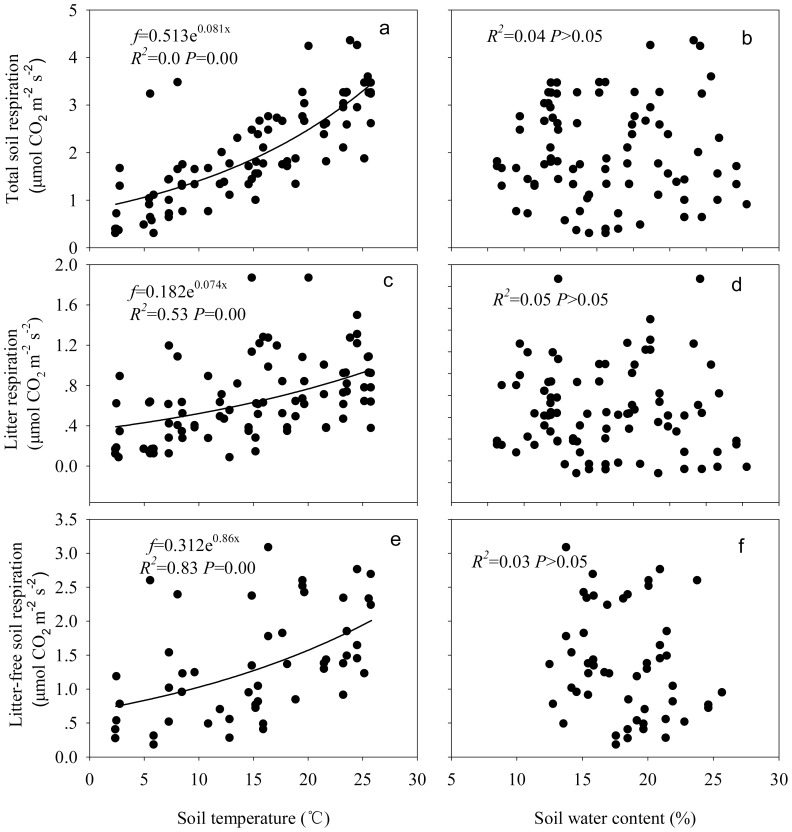
Relationships between the total soil respiration (a, b), litter respiration (c, d), litter-free soil respiration (e, f) and soil temperature at 5 cm depth or soil water content at 5 cm depth in different-aged *Pinus massoniana* forests during the 18-months field observations.

There were significant exponential relationships between soil temperature (*T*_5_) and the total soil respiration (*R* ^2^ = 0.80, *P*<0.001; Fig. 5a), litter respiration (*R* ^2^ = 0.53, *P*<0.001; Fig. 5c), or litter-free soil respiration (*R* ^2^ = 0.83, *P*<0.001; Fig. 5e). The corresponding *Q_10_* was 2.25 for the total soil respiration, 2.10 for the litter respiration, and 2.36 for the litter-free soil respiration. No clear relationships between respirations and soil water contents were found (Fig.5b, d, f).

The co-variant effects of soil temperature and moisture on soil respiration were significant for the three stands (*R* ^2^ = 0.70, 0.42, 0.69, all *P*<0.001; Table 4). There were no combined effects of soil temperature and soil or litter water contents on litter respiration (Table 4).

**Table 4:** The functions and model fit parameters (*n*, *R* ^2^ and *P*-values) between measured soil respiration with soil temperature (T), soil moisture content (SWC) and litter water content (LWC) in *Pinus massoniana* forests.

Stand	Equations	*n*	*R* ^2^	*P*
Total soil respiration in relation to T and SWC				
20-year-old stands	*R*s = 0.040e^0.072T^ (1.555SWC–0.037SWC^2^)	162	0.695	<0.001
30-year-old stands	*R*s = 0.060e^0.048T^ (1.729SWC–0.045SWC^2^)	162	0.420	<0.001
46-year-old stands	*R*s = 0.040e^0.062T^ (1.380SWC–0.017SWC^2^)	162	0.693	<0.001
Litter respiration in relation to T and SWC				
20-year-old stands	*R*s = 0.038e^0.051T^ (1.158SWC–0.035SWC^2^)	162	0.201	>0.05
30-year-old stands	*R*s = 0.042e^0.002T^ (1.618SWC–0.049SWC^2^)	162	0.031	>0.05
46-year-old stands	*R*s = 0.020e^0.047T^ (1.106SWC–0.007SWC^2^)	162	0.240	>0.05
Litter respiration in relation to T and LWC				
20-year-old stands	*R*s = 0.039e^0.047T^ (0.841LWC–0.019LWC^2^)	162	0.163	>0.05
30-year-old stands	*R*s = 0.052e^0.002T^ (0.564LWC–0.017LWC^2^)	162	0.157	>0.05
46-year-old stands	*R*s = 0.049e^0.054T^ (0.603LWC–0.011LWC^2^)	162	0.256	>0.05

**Figure 6:**
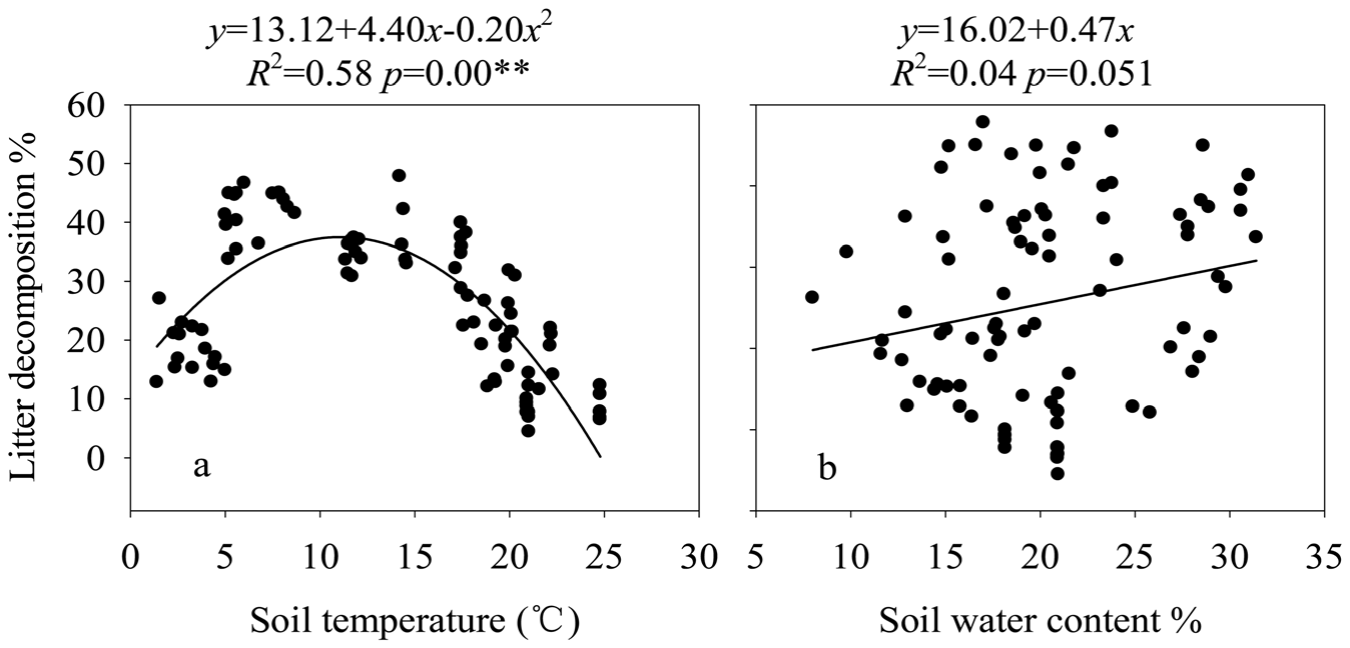
Relationships between litter decomposition (%) and soil temperature (a) or soil water content (b) in different-aged *Pinus massoniana* forests.

Soil moisture was found to be marginally significantly linearly correlated with litter decomposition (*R* ^2^ = 0.043, *P* = 0.051; Fig. 6b). The litter decomposition was significantly quadratically correlated with soil temperature (*R* ^2^ = 0.58, *P*<0.001; Fig. 6a). The temperature sensitivity (*Q*_10_ value) of litter mass loss was 1.5.

## Discussion

### Effects of forest age on soil and litter respiration

Ewel et al. [31] suggested that soil respiration decreased with forest age in temperate forests, while it increased with forest age in tropical and subtropical forests. The present study found that forest age affected the litter respiration during the growing season and influenced the litter-free soil respiration and the total soil respiration during the dormancy period (Table 3). Our results suggest that the effects of forest age on soil respiration may be mainly caused by stand roots in terms of root quantity (roots biomass) and quality (fine roots fraction) which increase with increasing forest age [32]. Previous studies indicated that forest age had a significantly positive influence on soil respiration in *Pinus taeda* plantations [33] and in warm-temperate oak forests [32]. Tang et al. [34] and Bolstad et al. [35] found that soil respiration first increased and then decreased with increasing forest age. Saiz et al. [36] found that soil respiration decreased with forest age during the younger stages of Sitka spruce forests. These results indicate that the effects of forest age on soil respiration differ with tree species and stand structure probably associated with the amount of fine roots and the quality of soil C pools [36].

Our results showed that the forest age effect on litter and soil respiration was nonlinear. For example, the mean litter respiration rate was 0.66, 0.54, and 0.74 µmol CO_2_ m^−2^ s^−1^ for the 20-, 30-, and 46-year-old stands, respectively. This lower litter respiration in the 30-year-old stands seemed to be a result of the lower soil organic matter and nitrogen contents (Table 1), which may have led to decreases in soil microbial activities [37]. The lower litter respiration in the 30-year-old stands may also be resulted from the lower initial litter quality compared to that in the other two stands (Table 2) because litter quality directly affects litter decomposition rates [1]. Hättenschwiler and Gasser [38] showed that the decomposition rate was significantly positively correlated with litter N concentration (*R* ^2^ = 0.75, *P* = 0.025) but negatively correlated with litter C/N ratio (*R* ^2^ = 0.72, *P* = 0.032) and lignin/N ratio (*R* ^2^ = 0.75, *P* = 0.026). Bini et al. [39] found that plant litter with a C/N ratio of <25 degraded easily. Fanin et al. [40] reported that litter substrate quality (N, P content) was the most important factor explaining the observed spatial variations in soil respiration, and higher respiration rates were associated with high litter N and P contents.

Saiz et al. [36] indicated that the interactive effects of abiotic and biotic factors may modify the influences of forest age on soil respiration. Previous studies have reported both increased [41] and decreased [42] decomposition rates along a chronosequence of forest stands. Our present study found that litter decomposition rates did not significantly differ with forest age (Table 3), this may be caused by the difference in soil nutrients among the three stands (Table 1). Our previous study with *P. massoniana* stands found that the decomposition rates were faster in soil nutrient-poor than in nutrient-rich stands [26]. Similarly, Barlow et al. [43] found that the secondary species (*Vismia* sp. and *Bellucia* sp.) decomposed slower than the primary forest (*Bertholettia* sp.) along successional stages. Pandey et al. [44] showed that natural Oak (*Quercus* sp.) forests had lower soil nutrient level and greater litterfall with faster decomposition rates compared to Oak plantation forests. These studies suggest that not only forest age but also other factors such as site conditions (e.g. soil nutrients) and litter quality interact to affect litter decomposition [26], [43].

### Effects of soil temperature and water contents on respiration and litter decomposition

Variation in soil temperature is one of the most important factors determining the seasonal and diurnal variations in soil respiration [17], and soil respiration is highly sensitive to changes in surface temperature [45]. The present study found that the significant effects of soil temperature on respirations can be well-described with simple exponential regression models (Fig5a, c, e), which support the results of Shi et al. [46] and Zimmermann et al. [1]. Fang and Moncrieff [45] showed that the responses of soil respiration to temperature were commonly described using exponential equations, and thus the temperature sensitivity of soil respiration (*Q*_10_) reduced at high temperature range. On the other hand, Davidson et al. [28] found that the temperature sensitivity of soil respiration (*Q*_10_) decreased with drought or water deficit. Luan et al. [47] suggested that both soil chemical and physical characters contributed to the *Q*_10_ variations. Hence, our study found that *Q*_10_ was 2.25 for the total soil respiration, 2.10 for the litter respiration, and 2.36 for the litter-free soil respiration.

Soil respiration is generally assumed to be strongly controlled by water availability [48]. In our study, we did not find any clear relationships between respirations and soil water contents (Fig. 5b, d and f). Previous studies indicated a wide range of relationships between respiration and moisture including linear [49], quadratic [50], exponential [28], logarithmic [51], and hyperbolic [52] relations, indicating that the physical (e.g.. diffusion), physiological (osmoregulation), and biochemical (enzyme dynamics) factors interact to affect the respiration-moisture relationships [48]. Recently, Moyano et al. [53] stated that further studies should concentrate on reducing uncertainties in the moisture-respiration relationships.

Soil temperature significantly interacted with soil water to affect the soil respiration (Table 4), it is probably because higher temperature may lead to lower soil water content and reverse [28]. Borken et al. [54] showed that the combined effects of temperature and water content on soil respiration were multiple linear regression. Lellei-Kovács et al. [55] showed that the interaction between temperature and moisture on soil respiration was straightforward for a linear model.

Davidson [28] found that soil temperature explained 80% of the variation of soil respiration, and there was no obvious relationship between soil water content and soil respiration. Peng et al. [56] pointed out that the sensitivity of soil respiration to temperature or moisture has not yet been adequately quantified, because most of the published results tend to be site-specific and no models have been widely accepted and commonly used.

Consistent with the results of Fiere et al. [20], we found that the effects of soil temperature on litter decomposition rates were quadratically well-described (*R* ^2^ = 0.58, *P*<0.001), which indicates that both lower or higher temperature lead to decreased decomposition rates (Fig. 6a). However, our results are inconsistent with the results of Aerts [57] who stated that warming resulted in increased decomposition rate.

Litter decomposition rates were found to positively respond to increased soil moisture [58]. In the present study, higher temperature resulted in lower soil moisture in the 30-year-old stands (Fig. 4). Higher temperature usually stimulates but lower soil moisture decreases the respiration and decomposition rates [55]. Cortze [21] and De Santo et al. [59] reported that soil moisture was most important during the early decomposition stage rather than the late stages. Under field experimental conditions, however, it is difficult to detect the net effects of moisture on respiration or decomposition because all factors interact to affect the litter decomposition and the effects of temperature might become stronger with increasing moisture [19].

Climate factors such as temperature and moisture alone and combined affect litter decomposition [26]. Butenschoen et al. [60] suggested that litter decomposition increased with increasing temperature in the high moisture treatment and decreased with increasing temperature in the low moisture treatment. Many studies failed to find positive responses of litter decomposition to warming when moisture is limited [57]. Cortez [21] reported that the relationships between litter decomposition rate and the ratio of soil humidity to temperature (H/T) showed a polynomial function (*y* = a*x* ^2^+b*x*+c), and soil temperature seemed to be the main determining factor in wet sites, while soil moisture was the most important factor during the early decomposition stages in dry period.

### Relationships between soil respiration and litter decomposition

Litter provides the major C source for soil respiration [6]. Subke et al. [7] showed that an increase in litter input promotes rhizosphere respiration and rhizosphere activity, leading to increases in soil respiration. Reynolds and Hunter [6] showed that the soil respiration was significantly reduced by litter removal. Increased litter input stimulated soil microbial activity and soil C loss by microbial respiration [61]. In our study, the total soil respiration (Fig. 3a) was positively quadratically correlated with litter decomposition rate, which indicates that the soil respiration rates may be reliable indicators for long-term litter decomposability and litter carbon dynamics. Similarly, Aerts and de Caluwe [23] reported that the litter respiration rates were positively correlated with litter mass loss rates.

We found that both litter decomposition rate and soil respiration had similar temperature sensitivity and both peaked in summer. These may imply that both soil respiration and litter decomposition in the present study are controlled by similar environmental factors [26]. Moreover, we found that litter respiration peaked nearly one month earlier than the litter-free soil respiration and the total soil respiration (Fig. 1), which may be partly caused by the seasonality of the microbial community composition [46] associated with the environmental conditions, as reported by Berryman et al. [62].

The addition or exclusion of fresh litter was found to significantly increase or decrease the total soil CO_2_ efflux, respectively [4], [61]. The present study found that the mean contribution of the litter respiration to the total soil respiration ranged from 31.0% (30-year-old stands) to 45.9% (20-year-old stands) (see also [1], [27]). Buchmann [16] estimated that soil respiration rates reduced by 10–20% when the litter and semi-decomposed litter layer were removed, and even reduced by up to 30-40% when the humus layer was also additionally removed.

The contribution of the litter respiration to the total soil respiration seems to vary with study area, tree species, and soil fertility [63]. Berger et al. [10] suggested that decomposing litter contributed 22–32% (base-rich sites) and 11–28% (base-poor sites) to the total soil respiration. In temperate coniferous forest ecosystems, the estimated contribution of leaf litter respiration to the total soil respiration exhibit a large seasonal variation from 2% (early spring) to 20% (mid summer) [4]. Cisneros-Dozal et al. [66] also found that the contribution of leaf litter decomposition to the total soil respiration increased from 5±2% (6±3 mg C m^−2^ hr^−1^) during a transient drought to 37±8% (63±18 mg C m^−2^ hr^−1^) immediately following water addition, indicating the effects of water availability on the contribution. In our study, the contribution of the leaf litter respiration to the total soil respiration was larger in winter than in summer. A possible explanation is that lower winter temperature limits the root respiration, and the variation of litter respiration is less important to the total soil respiration than the root respiration does [63]. Therefore, the soil respiration is obviously affected by many biotic and abiotic factors including root biomass and root activity across time [64].

In conclusion, the present paper revealed that soil moisture and temperature play complex roles in determining the respiration and decomposition across spatial and temporal scales in the *P. massoniana* forests studied. The apparent temperature sensitivity of soil respiration and litter decomposition is influenced not only by soil water but also by a wide range of factors including soil nutrients and litter quality [55], [65]. The contribution of litter decomposition to the total soil respiration varies across spatial and temporal scales. These findings emphasize the need for separate consideration of soil and litter respiration when assessing climate impacts on forest carbon cycling [62].
